# Time-Frequency Multi-Domain 1D Convolutional Neural Network with Channel-Spatial Attention for Noise-Robust Bearing Fault Diagnosis

**DOI:** 10.3390/s23239311

**Published:** 2023-11-21

**Authors:** Yejin Kim, Young-Keun Kim

**Affiliations:** 1 Department of Mechanical and Control Engineering, Handong Global University, Pohang 37554, Republic of Korea; yjkim@handong.ac.kr; 2 School of Mechanical and Control Engineering, Handong Global University, Pohang 37554, Republic of Korea

**Keywords:** multi-domain fusion, bearing fault diagnosis, PHM, attention, noise robustness, fault classification

## Abstract

This paper proposes a noise-robust and accurate bearing fault diagnosis model based on time-frequency multi-domain 1D convolutional neural networks (CNNs) with attention modules. The proposed model, referred to as the TF-MDA model, is designed for an accurate bearing fault classification model based on vibration sensor signals that can be implemented at industry sites under a high-noise environment. Previous 1D CNN-based bearing diagnosis models are mostly based on either time domain vibration signals or frequency domain spectral signals. In contrast, our model has parallel 1D CNN modules that simultaneously extract features from both the time and frequency domains. These multi-domain features are then fused to capture comprehensive information on bearing fault signals. Additionally, physics-informed preprocessings are incorporated into the frequency-spectral signals to further improve the classification accuracy. Furthermore, a channel and spatial attention module is added to effectively enhance the noise-robustness by focusing more on the fault characteristic features. Experiments were conducted using public bearing datasets, and the results indicated that the proposed model outperformed similar diagnosis models on a range of noise levels ranging from −6 to 6 dB signal-to-noise ratio (SNR).

## 1. Introduction

Bearing faults, which account for up to 50% of rotating machinery failures [1], are critical concerns in industrial operations. The consequences of undetected bearing failures in their early stages can lead to significant economic losses within industrial facilities. However, directly diagnosing the health condition of bearings is challenging because they are concealed from visual inspection and are tightly coupled to the other components of rotating machinery. A promising solution to bearing health monitoring without visual inspection is the analysis of vibration signals from rotating machinery [2,3]. The vibration signals of the faulty bearings exhibit a distinct behavior characterized by abnormal impulse signals, which occur by collision events of the rolling elements in a damaged area. By extracting and analyzing the signal features that represent the characteristics of bearing faults, the bearing health stages can be classified.

Data-driven bearing monitoring systems using machine learning models have been extensively studied and applied in industry [4,5,6,7]. Machine learning models such as the support vector machine [4,5], principal component analysis [8], k-nearest neighbors [6], and random forest [7,9] were widely implemented for bearing fault classification models. Most of the bearing diagnosis models are designed to classify the single type of bearing faults. Some studies attempted to design a model that diagnoses bearings with compound faults [10,11].

However, classical machine learning methods have weaknesses against unexpected factors, including disturbance, noise, and variation in the operation conditions.

Deep learning-based models typically outperform machine learning methods due to their deep and advanced network architectures [12] that are trained with vast datasets. A notable advantage of deep learning is that it obviates the explicit feature extraction and selection processes from the input data. Deep learning models excel in extracting complex and nonlinear features from the dataset for precise and efficient data analysis. For these reasons, deep learning models are more suitable for analyzing complex bearing fault signal datasets that can be corrupted by various types of noise and disturbances [13]. These models aim to autonomously extract failure-related features solely from vibration signals without any human manual intervention. Yu et al. [14] proposed an autoencoder-based network to overcome the limitations of the drop in the accuracy of the machine learning model in changeable operating conditions. Zuo et al. [15] applied a spiking neural network structure composed of biological neurons for bearing fault diagnosis with a small amount of dataset. Jin et al. [16] proposed a residual network with an attention module that can detect bearings with compound faults.

For a fast and light-weight classification model for bearing faults, one-dimensional convolutional neural network (1D CNN) models that directly process the time domain vibration signals have been proposed in the literature [17,18]. Several variants of time domain 1D CNN models for bearing diagnosis have been reported, such as 1D CNN with long short-term memory [19] and 1D CNN model with a multi-scaled information fusion layer [20]. Ji et al. resampled vibration signals obtained from a variable rotation speed system into constant speed signals using an order-tracking algorithm and applied them to a 1D CNN to extract features adaptively [21]. Some studies have been conducted to design a 1D CNN-based bearing fault diagnosis model that is light enough to be deployed in an embedded system [22,23]. Ding et al. proposed lightweight CNN with parameter transplantation that can be easily embedded in low-cost MCUs [22]. Most 1D CNN models that use raw vibration time signals can face challenges when the signals are masked by resonances, noise, and other disturbances. Furthermore, in contrast to conventional approaches that use frequency domain analysis, using the time domain signal is challenging for understanding the process to obtain results for humans.

In contrast, the frequency domain of the vibration signal offers several advantages in distinguishing the unique vibration characteristics for each fault class [24,25]. Additionally, the fault spectrum can be separated from noise in other frequency bands to enhance robustness against noise [2]. For these reasons, using frequency domain features makes it easy to understand and analyze the decisions made by the model with explainable artificial intelligence (XAI) tools.

However, there are several factors relating to bearing faults that may not be revealed through frequency analysis. For example, bearing faults at the earliest stage results in a weak high-frequency or resonance signal [26]. During this stage, vibration signals due to friction result from the resonance, which is not directly related to bearing cracks. For this reason, some fault-related data might be lost while converting the time domain raw signal into the frequency domain, so it is not recommended to exclude the time domain signal completely.

Two-dimensional (2D) CNN utilizing a time-frequency image as the input, one prevalent approach for applying frequency domain analysis to CNNs, has been studied extensively. These models use both temporal and spatial information in order to enhance the comprehensiveness of the input [27]. Widely used 2D conversion methods for bearing signals include the wavelet packet energy [28], short-time Fourier transform (STFT) [29], symmetrized dot pattern (SDP) [30], Cyclic Spectral Coherence (CSCoh) [31], and continuous wavelet transform (CWT) [32,33]. Ruan et al. [34] designed 2D CNN network parameters based on fault signal analysis such as fault characteristic frequency for bearing fault diagnosis. Du et al. [33] preprocessed the bearing fault signal with CWT and selected the important frequency range for the 2D CNN model using the explainable deep learning technique to diagnose the bearing fault. Like 1D freq CNN, it allows for the interpretation of the model’s output results by humans. The reasonableness of the decision-making process of the model can be evaluated by analyzing the frequency domain utilized for its output [29]. However, the 2D CNN approach requires a relatively higher calculation load, which has the disadvantage of applying the model to a low-power computer.

Recently, mixing both time and frequency domain signals on 1D CNN architecture has been proposed. Sun et al. proposed the domain fusion 1D CNN having multi-channel input composed of the raw signal, envelope spectrum, and discrete cosine transform [35]. It does not process each domain individually but applies a shared kernel to input features, channel-wise concatenated multi-domain input. A multi-domain parallel CNN that operates individually for each domain was proposed [36]. It employs raw signals as the time domain and short-time Fourier transform (STFT) images as frequency domain input. Dong et al. [37] designed multi-domain feature fusion CNN. Input features for the model are manually extracted features, including root mean square and peak-to-peak.

This paper also proposes a multi-domain fuse model that extracts both the frequency domain and time domain features for a high performance in the bearing-fault diagnosis. The model effectively extracts representations from both the time domain, containing complicated high-dimensional features of fault signals, and the frequency domain, containing distinct fault characteristic features, to extensively comprehend the bearing fault signals.

Compared with existing approaches, this study is focused on simpler but highly effective multi-domain 1D CNN models. Unlike the previous studies, it does not calculate the statistical features nor uses the STFT processing. Furthermore, instead of combining the multi-domain signals at the input layer, the proposed model extracts each domain feature independently to acquire broader representations at different domains.

Furthermore, this study is aimed to improve the noise robustness of bearing diagnosis to be implemented under harsh environments. The proposed model applied an attention mechanism to help the model focus more on the important fault components and pay less attention to other meaningless signals such as noise. The effectiveness of attention modules in bearing diagnosis has been validated in several pieces of literature. Wang et al. converted bearing vibration signals into 2D gray images and applied multi-head attention to increase CNN model accuracy of bearing fault diagnosis [38]. Wang proposed an SDP image representation used as input to a model for bearing fault diagnosis and used SENet, one of the most popular channel attention mechanisms [30]. Huang et al. [39] improved performance under noisy environments by applying channel attention to 1D vibration signals. Hao et al. [40] proposed a multi-scale and attention mechanism-based 1D CNN and PLakias employed attention-dense 1D CNN for fewer network parameters [41]. This study applied a combination of channel attention and spatial attention in sequence [42] to focus on important sub-areas of feature maps for overall noise robustness and accuracy.

In summary, this paper proposes a time-frequency multi-domain 1D CNN with attention modules (TF-MDA) for accurate and robust bearing fault monitoring. The main contributions are as follows:

1. It proposes a time-frequency multi-domain feature extraction and fusion model for accurate bearing fault diagnosis.
2. The proposed model has a simple but effective 1D CNN architecture for low overhead. Furthermore, it uses minimum preprocessing of bearing-physics-informed envelop extraction and fast Fourier transform.
3. The proposed model applied channel-wise and spatial-wise attention modules that enhanced the overall noise-robustness to be implemented under a strong noise and disturbance environment.

The remainder of this paper is organized as follows. In Section 2, background knowledge to understand the methods used in the proposed model is explained. The preprocessing and the proposed model are explained in detail in Section 3. The experimental results and analysis are described in Section 4. Finally, conclusions are drawn in Section 5.

## 2. Background

### 2.1. Frequencies of Bearing Fault Signals

Ball bearings are composed of inner race, outer race, ball, and cage elements. When damaged elements are in a rotating bearing, periodic fault characteristic signals are generated. The bearing fault signals have unique frequencies that are determined by the mechanical parameters of the bearing and the fault locations. The fault types of a ball bearing in the implemented datasets are classified as the inner race fault, outer race fault, and ball fault. The fault frequencies for each bearing fault type can be estimated using the following formula [43]:

Ball pass frequency for inner race (BPFI):

(1) $$\begin{array}{c} f_{IR}=\frac{Z\left(1\;+\;\cos\;\alpha \;\times \;\frac{d}{D}\right)}{2}\times f_{r} \end{array}$$

Ball pass frequency for outer race (BPFO):

(2) $$\begin{array}{c} f_{IR}=\frac{Z\left(1\;-\;\cos\;\alpha \;\times \;\frac{d}{D}\right)}{2}\times f_{r} \end{array}$$

Ball spin frequency (BSF):

(3) $$\begin{array}{c} f_{BS}=\frac{1}{2}\frac{D}{d}\left(1-{\left(\frac{d}{D}\times \cos\alpha \right)}^{2}\right)\times f_{r} \end{array}$$

where $f_{r}$ is the shaft rotational speed, *Z* is the number of rolling elements, *D* is the pitch circle diameter of the bearing, *d* is the rolling element or ball diameter, and α is the contact angle.

The domain knowledge of the fault characteristics is implemented in the signal processing of bearing datasets.

### 2.2. Envelope Extraction

A defect bearing generates impact forces as the rolling elements interact with the damaged area. The time domain vibration from a defect bearing is a mixture of fault characteristics signals modulated to the shaft resonance. Furthermore, the vibration signal contains noise and background vibration from other machinery. Thus, a series of signal preprocessing is necessary to highlight the bearing fault characteristic features from the complex signals.

Envelope extraction is a preprocessing method that extracts the bearing fault characteristic signals from the raw signals of the amplitude-modulated to the resonance of shaft rotation. The relative lower frequency components of bearing fault characteristics can be emulated from the other high-frequency variations by applying the Hilbert transform. The Hilbert transform is a method that computes the analytic signal of the real-valued bearing vibration. The magnitude of the analytic signal is the envelope of the raw signal.

Mathematically, the Hilbert transform shifts the signal phase by π/2 while maintaining the amplitude. The analytical signal ($x_{h}$) can be defined as the sum of the original signal and its Hilbert transform with the mathematical expression of

(4) $$\begin{array}{c} x_{h}\left(t\right)=x\left(t\right)+j\hat{x}\left(t\right) \end{array}$$

where $x\left(t\right)$ is original signal, and $j\hat{x}\left(t\right)$ is Hilbert transform of $x\left(t\right)$.

**Figure 1:**
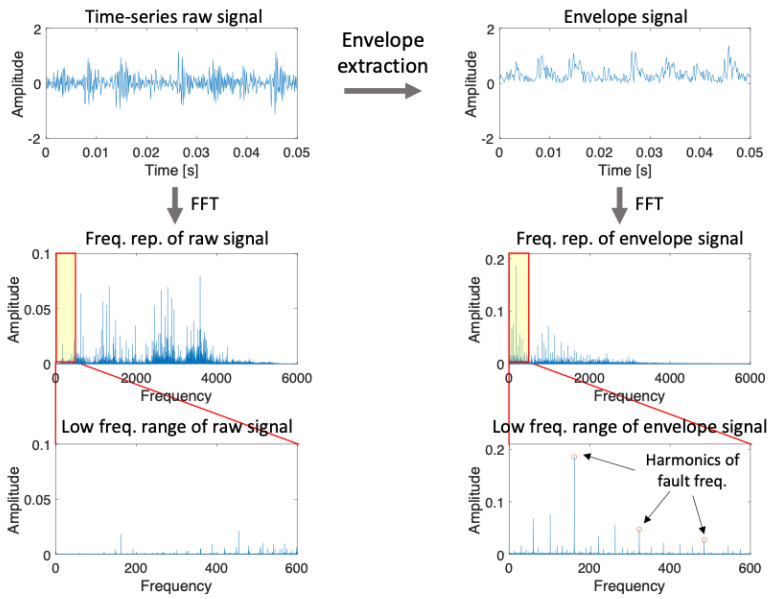
Envelope extraction and FFT of the CWRU bearing vibration signal.

In this manner, the envelope signal can be extracted from the amplitude-modulated signal at the resonance, as illustrated in Figure 1, which is the absolute value of this analytical signal, $\left|x_{h}\left(t\right)\right|$. When comparing the frequency domain of the raw signal with that of the envelope-extracted signal, the latter has a more distinct fault characteristic frequency than the former.

### 2.3. Convolutional Block Attention Module (CBAM)

The attention mechanism helps the network focus on salient parts of the features for improving performance. It is applied extensively in natural language processing to allow the seq-to-seq model to focus on the part related to the current step while grasping the entire context in machine translation of natural language [44]. Recently, the attention concept has been applied to image processing with CNN models to focus more on highly meaningful features [45].

**Figure 2:**
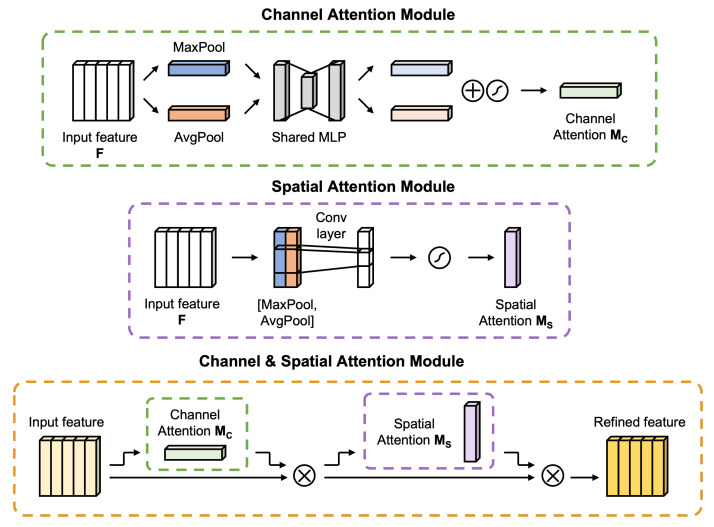
Diagrams of each attention sub-module and the entire module.

The CBAM [42] is a hybrid attention module composed of a sequence of channel attention and spatial attention and can be easily inserted into a 2D CNN model to enhance the network representation power. Since the proposed model has 1D CNN architecture, the applied CBAM is modified accordingly, as illustrated in Figure 2.

This attention module can focus on the salient parts of the input features by integrating two-direction information: cross-channel and spatial. The average pooling and max pooling are applied in the channel and spatial directions to exploit a wider range of information for improving the feature representation. After the results are summed element-wise, the overall result is nonlinearized using the sigmoid function and multiplied by the original input convolutional block.

For the channel attention module, max pooling and average pooling are applied across the channels of the input feature map. These two output vectors are combined with the element-wise summation by a weight-shared multi-layer perception. The mathematical expression for channel attention is as follows:

(5) $$\begin{array}{c} M_{C}\left(F\right)=\sigma \left(MLP\left(AvgPool\left(F\right)\right)+MLP\left(MaxPool\left(F\right)\right)\right)\\ =\sigma \left(W_{1}\left(W_{0}\left(F_{avg}^{c}\right)\right)+W_{1}\left(W_{0}\left(F_{max}^{c}\right)\right)\right) \end{array}$$

where *F* is the input feature block, σ symbolizes the sigmoid function, MLP is the weight-shared multi-layer perceptrons (MLP), $W_{0}\in R^{C/r\times C}$, *C* is the number of channels, *r* is reduction ratio, and $W_{1}\in R^{C\times C/r}$ are shared MLP weight. Two vectors of size c×1 are made by channel-wise average and max pooling the feature map. They are, respectively, input into the weight-shared MLP. The MLP has a structure that reduces a vector of length c by a reduction ratio *r*, to c/r size vector, and then stretches its length to *c*.

Similarly, the spatial attention is concatenated after average and max pooling in the spatial direction and integrated into a single channel through convolution as

(6) $$\begin{array}{c} M_{S}\left(F\right)=\sigma \left(f^{N\times 1}\left(\left[AvgPool\left(F\right);MaxPool\left(F\right)\right]\right)\right)\\ =\sigma \left(f^{N\times 1}\left(\left[F_{avg}^{s};F_{max}^{s}\right]\right)\right) \end{array}$$

where $f^{N\times 1}$ represents a convolution operation with the kernel size N×1.

The sequence of applying the channel attention and then spatial attention generates the output feature map of $F^{\prime \prime }$ from the input feature *F* through

(7) $$\begin{array}{c} F^{\prime }=M_{C}\left(F\right)⊗F,\\ F^{\prime \prime }=M_{S}\left(F^{\prime }\right)⊗F^{\prime } \end{array}$$

where ⊗ denotes the element-wise multiplication, *F* depicts the intermediate feature map, $M_{C}$ indicates the channel attention map, and $M_{S}$ represents the spatial attention map.

## 3. Proposed Network Model

### 3.1. Proposed Architecture Overview

**Figure 3:**
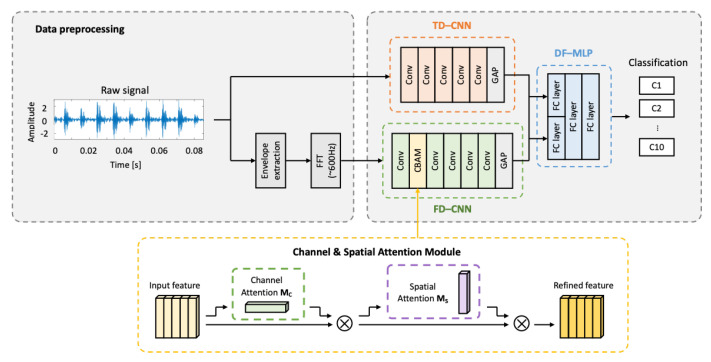
Overview of the proposed TF-MDA model.

This paper presents a time-frequency multi-domain 1D CNN with attention modules (TF-MDA) for a noise-robust bearing fault monitoring system, which is described in Figure 3. The proposed network is composed of three main networks: the time domain CNN (TD-CNN), frequency domain CNN (FD-CNN), and multi-domain fusion network with the classification head, as shown in Figure 3.

The time domain CNN (TD-CNN) model is designed to extract implicit high-dimensional features in raw vibration signals of bearing, which is challenging for direct interpretation. Frequency domain CNN (FD-CNN) extracts fault features from the frequency domain signals preprocessed from the raw vibration signals. The frequency domain analysis of bearing signals is easier to interpret to identify the fault characteristics at the expected fault frequencies, as explained in Section 2. The features extracted from each network are fused in a fully connected layer, followed by the classification head of multi-layer propagation layers to diagnose the bearing conditions.

### 3.2. Data Preprocessing

#### 3.2.1. Dataset Augmentation

The proposed model was designed based on the bearing datasets of the Case Western Reserve University (CWRU) benchmark [46], which is explained in detail in Section 4.

The input data are the time domain acceleration signals measured on the bearing bracket. To train the proposed deep model with sufficient datasets, a data augmentation method was implemented to increase the dataset numbers.

**Figure 4:**
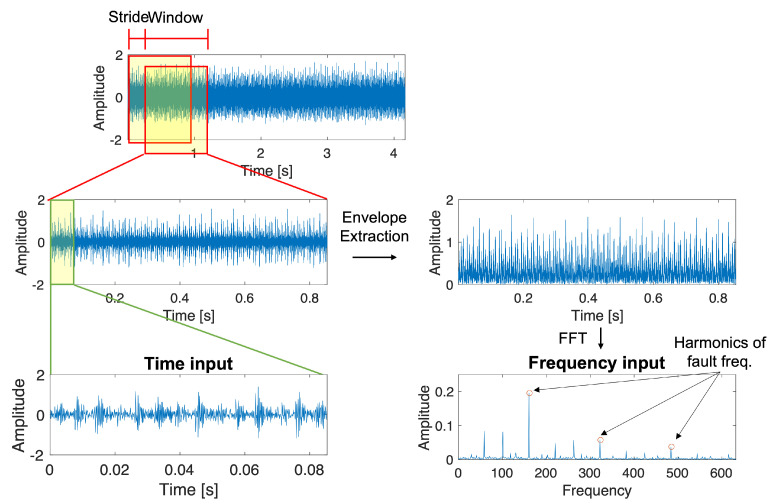
Overview of preprocessing time domain and frequency domain signals.

This study used the overlapping window to increase the training data samples, as illustrated in Figure 4. The appropriate frequency resolution for the bearing fault characteristics was considered to determine the length of the overlapping window. The frequency resolution is the ratio of the sampling frequency and the window length as

(8) $$\begin{array}{c} f_{res}=f_{sampling}/L_{window} \end{array}$$

The sampling rate of the raw vibration signal is 12 kHz for 10 s. The bearing fault frequencies up to the 3rd harmonic are within 600 Hz; it is decided that the frequency resolution of 1 Hz is adequate in analyzing the fault signal spectrum. Thus, to have about 1 Hz of frequency resolution for the frequency domain data, the window length of 10,240 points was chosen. These augmented signals of each sliding window were then passed to the further processing of envelop extraction for the frequency domain network.

For the input of the time domain network, the augmented signals were further oversampled to decrease the data point numbers from 10,240 to 1024 for each datum, as shown in Figure 4. The oversampled data in the time domain contain about 8 to 9 periodic fault signals, which can be considered sufficient. This signal of 1024 points is passed directly to the time domain network (TD-CNN) without further processing.

#### 3.2.2. Envelope Extraction and Fast Fourier Transform

The augmented dataset was processed to enhance the visibility of fault characteristics before being passed to the input of the frequency domain network (FD-CNN).

As explained in the previous section, the fault characteristic signals are modulated in amplitude with the resonance frequency of the shaft [2]. The harmonics of the affected frequency can be demodulated through the envelope extraction process, which helps in extracting the fault signals, as shown in Figure 1.

The envelope signals are then transformed to the frequency domain spectrum with the fast Fourier transform (FFT). The resulting FFT spectrum has a range from 0 Hz to 6 kHz. However, the important fault characteristic frequencies are mainly concentrated in low-frequency regions. Therefore, the FFT spectrum was cropped to 600 Hz, which is the frequency range of fault frequencies up to the 3rd harmonics. The final processed data for the frequency domain input have a length of 520 points.

The frequency spectrum of the bearing vibration signals provides essential features and information on the bearing defects and noise, which are clearly visible at the fault frequency harmonics. In contrast, the time domain vibration signal is a highly complex signal to analyze the fault characteristics. However, it contains comprehensive time-series feature representations for bearing condition classification.

Thus, the proposed network fuses the features extracted from the time domain and frequency domain networks for a richer feature representation of the bearing defect signal than would be possible by applying a single-domain signal. Furthermore, the proposed model is based on 1D-CNN architecture, which has a smaller parameter overhead than 2D-CNN-based models that use 2D images of time-frequency signals from STFT (short-time-frequency transformation).

### 3.3. Multi-Domain Feature Extraction

#### 3.3.1. Time Domain Feature Extraction Network

The input of 1024 points from the time series signals is passed to the designed 1D CNN (TD-CNN) to extract time domain features. The TD-CNN has a simple architecture for a lightweight network but has adequate feature extraction layers. A series of five convolutional layers with the rectified linear unit (ReLU) activation function and batch normalization are used for high-level feature extraction. In each layer, the convolution strides are applied to reduce the feature resolution while preserving useful local representations [47].

After all the convolutional layers are processed, the resulting feature map is passed to the global average pooling (GAP) layer that generates a flattened 1D feature vector. GAP is selected because it preserves the global context in the features, is robust to spatial translation of the input, and alleviates overfitting by reducing the number of trainable parameters [48].

#### 3.3.2. Frequency Domain Feature Extraction Network

The frequency domain 1D CNN (FD-CNN) extracts the features of the frequency spectrum in parallel to TD-CNN. Similarly to TD-CNN, a series of five convolution layers are processed with strides, ReLU activation function, and batch normalization. Global average pooling is applied to the final feature map to generate the flattened 1D feature vector in the same size as the time domain block output.

### 3.4. Multi-Domain Feature Fusion

The 1D feature maps from each time and frequency domain fracture extractions are concatenated in the domain fusion network. The concatenated vector is passed to a series of fully connected layers of MLP layers to integrate the multi-domain representations to classify the bearing faults. The final layer is the softmax, in which output nodes are selected according to the number of bearing fault classes.

### 3.5. Attention Module for Noise Robustness

Attention modules are inserted in the network to highlight essential feature regions to improve classification performance and noise robustness. The bearing signals from actual industry sites can be corrupted with various types of noise depending on the working environment. Thus, the proposed model implemented a lightweight attention module to improve the robustness of signal noise as well as the overall classification performance.

The attention module for the proposed model is designed to extract the global and local features of the input data. Thus, channel-wise and spatial-wise attention modules [42] were applied in the frequency domain feature extractions as shown in Figure 3. In the spectral signal, each different fault type has its distinctive frequencies that are distinguishable. In contrast, it is hard to distinguish the signals of each fault type in the time domain features. Therefore, this study applied the attention module only in the frequency domain to highlight the representations of fault frequencies for bearing classifications.

The attention layer was inserted after the first convolutional layer in FD-CNN to ensure the network extracts the appropriate frequency domain representations at the low-level features. To determine an appropriate location to insert the attention layer, the model accuracies for each location of the attention layer were compared as shown in Table 1. Accuracy was similar in all layers, and placing the attention layer after the first convolutional layer showed the best performance. From an ablation study conducted, it is found that placing the attention module after the first convolutional layer results in the highest performance.

## 4. Experiment and Analysis

### 4.1. Network Parameters

The vector of 1024 length is passed to the TD-CNN first layer that operates convolution with 256 size wide kernel. Along the convolution layers, the kernel size is reduced to 15, 7, 7, and 3, whereas the feature channels are doubled starting from 4 channels. At the last layer of TD-CNN, the feature map is flattened by global average pooling.

FD-CNN has a similar architecture as TD-CNN, except it uses the frequency domain input of 540 length. In addition, attention modules are inserted after the first convolutional layer to emphasize important features at the early stage of feature extraction. The kernel size of the spatial attention is 64, whereas the channel attention has a reduction ratio (*r*) of 4.

After the attention module, frequency domain features are further extracted through four convolutional layers, and then the output feature map is flattened by global average pooling. The 64 length flattened vectors from each domain extraction are then concatenated to form a 128 length vector. Then, a series of MLP with dimensions of [124, 64, 32, 10] are processed to classify the bearing fault classes. The detailed network parameters are described in Table 2.

The cross-entropy loss function was used, and the Adam optimizer was applied with an initial learning rate of 0.001 in training the proposed network. Additionally, batch normalization was applied after every convolution to prevent over-fitting. The model was built and evaluated using the PyTorch framework. The maximum number of epochs was set as 20, and the training was stopped early if the validation accuracy continued to decline to prevent over-fitting. To suppress the impact of the data randomness, models were trained and tested five times, and the median value was used.

The dataset for training and testing the model was normalized according to the data type. The time domain vibration data were normalized by using the min–max normalization. For the frequency domain signal input for FD-CNN, the z-score normalization was used.

### 4.2. Case Study 1: Case Western Reserve University (CWRU) Dataset

#### 4.2.1. Dataset Description

The proposed model was trained for bearing diagnosis using the open dataset by the Bearing Data Center of CWRU [46]. It is a set of acceleration data of normal and faulty bearings that were acquired on a test bench of 2 HP motor machinery. The acceleration signal of the bearings was measured at the fan and drive ends of the motor at a 12-kHz sampling rate for approximately 10 s. Vibration signals were acquired using a 16-channel DAT recorder. The test bearings were deep groove ball bearings (SKF 6205-2RS) classified according to the bearing fault status: normal, inner-race fault, outer-race fault, and ball-rolling fault. These three fault classes were further divided by their defect sizes. For each fault size, working load conditions ranging from 0 to 3 HP were applied.

Using the bearing mechanical specifications, the fault characteristic frequencies for each fault type can be estimated as summarized in Table 3. The rotational frequency is approximately 30 Hz, and all the primary fault frequencies are within 165 Hz.

The bearing defect sizes of 0.007, 0.014, and 0.021 inches were selected for the fault classes of the inner race, outer race, and ball, respectively. For each fault class, the load conditions were selected from 0 to 3 HP. Thus, there were a total of 10 classes for the bearing health state, as shown in Table 4: the normal class and fault classes from three fault types (inner race, outer race, and ball) with three different fault sizes (0.007, 0.014, and 0.021 inches).

For each class, 860 samples were prepared from different load conditions selected evenly. Thus, the total number of samples was 8600, and they were partitioned at a ratio of 6:2:2 for training, validation, and testing, respectively. The numbers of samples used for training, validation, and testing were 5160, 1720, and 1720, respectively, and the data in each set were distributed evenly among the classes and working loads.

#### 4.2.2. Comparison Model

The following models were selected for comparison: WDCNN [49], TD-CNN, FD-CNN, TF-MDA(CSA). WDCNN [49] is 1D CNN model whose input is a time domain vibration signal. It is often used as a comparison model in several bearing diagnosis studies. MCNN-LSTM [19] extracts low- and high-frequency features using multi-scale kernels using 1D CNN model and then processed further with LSTM models. TD-CNN is the 1D CNN model for the time domain vibration signal and FD-CNN is the 1D CNN model for the frequency domain spectrum, which is part of the proposed model.

#### 4.2.3. Experiment Results under No Noise

First, the effect of the attention modules on the classification accuracy is analyzed to select the most appropriate attention type: no attention (NA), only channel attention (CA), only spatial attention (SA), and both channel and spatial attention (CSA). All these model variants were trained and tested under no-noise conditions. The classification accuracy was compared in Table 5.

Since all the model variants achieved similar prediction accuracy, the influence of the attention modules was not easily noticeable. Adding attention modules to the naive model increased the parameter numbers slightly by approximately 3%.

The proposed model, TF-MDA (CSA), was compared with similar bearing diagnosis models under the same conditions. All the comparison models were trained with the same dataset without noise. Their accuracies and numbers of trainable parameters are analyzed in Table 6. Results showed that the proposed model achieved the best performance with an accuracy of 100%. The compared models of WDCNN and FD-CNN also exhibited almost 100% performance.

To analyze the effectiveness of the feature extraction of the proposed model, t-distributed stochastic neighbor embedding (t-SNE) is used to visualize the feature clusters at the first and the last convolution layers in the frequency domain block. The method of t-SNE is a process that projects a high-dimension feature map to a two-dimensional plot for visualization. The feature distributions among the same and different fault classes are useful information explaining the effectiveness of classification accuracy. Ideally, the between-class variance should be high whereas within-class variance should be low for clear separability among fault classes.

**Figure 5:**
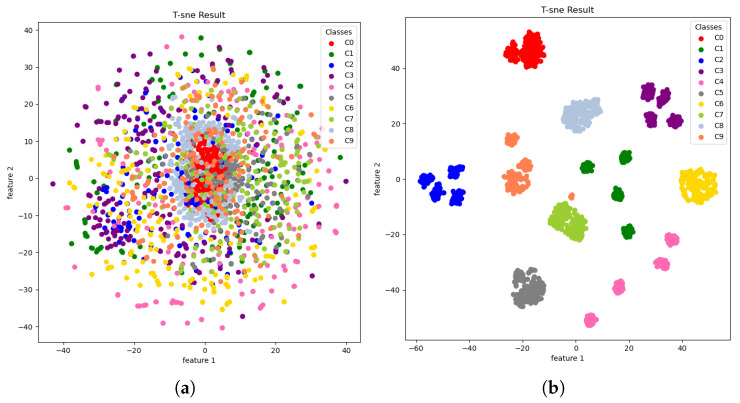
t-SNE: dimension-reduced features extracted from TF-MDA (CSA): (**a**) first convolutional layer; (**b**) fourth convolutional layer.

The results of t-SNE plots are shown in Figure 5, and the bearing fault classes are displayed in different colors to illustrate the feature separability. The t-SNE plot at the first convolutional layer indicated that the feature clusters of different fault classes are distributed largely and mixed all together, making it difficult to derive class separation boundaries. In contrast, at the fourth convolutional layer, the different class features are observed to have clearer boundaries while the same-class features approach each other. This suggests that the fourth convolutional layers were enough to have clear separation among different classes, reducing the misclassification error.

#### 4.2.4. Experiment Results under Random Noise

The model variants with different attention modules were analyzed with the test datasets with noise added to evaluate their robustness. The test data were mixed with Gaussian white noise of various levels, with signal-to-noise ratios (SNRs) ranging from −6 to 6 dB. The SNR is defined as the ratio of the signal power ($P_{s}$) to the noise power ($P_{n}$), i.e., $SNR=\frac{P_{s}}{P_{n}}$. For each noise level, the normalization was performed on the test dataset as explained in Section 4.1.

**Figure 6:**
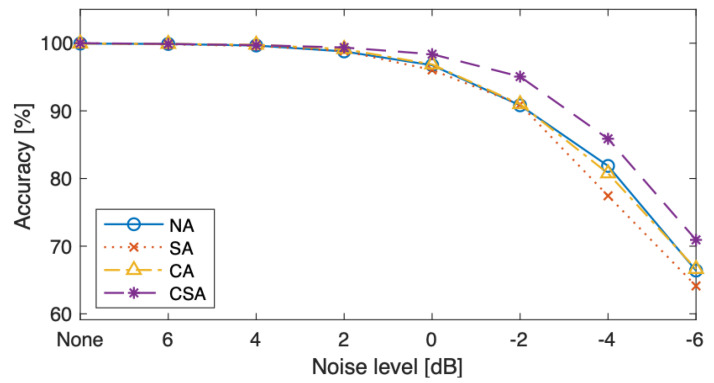
Classification accuracy without and with various noise conditions for different attention modules [%].

The classification accuracies under the various noise levels are compared in Table 7. Figure 6 shows classification accuracies under all conditions, without and with noise. As the noise level was increased from 2 dB to −6 dB, using both the spatial and channel attention modules in TF-MDA (CSA) exhibited the highest accuracy. At the noise level of 0 dB, when the signal and noise levels were the same, the accuracy of the TF-MDA (CSA) model was reduced from 100% to 97.15% (by 2.85%). In the case of noise level −6 dB, the TF-MDA (CSA) could exhibit the least performance degradation. The experimental results confirmed that using both channel and spatial attention is the most effective approach for improving the model performance under strong noise conditions.

**Figure 7:**
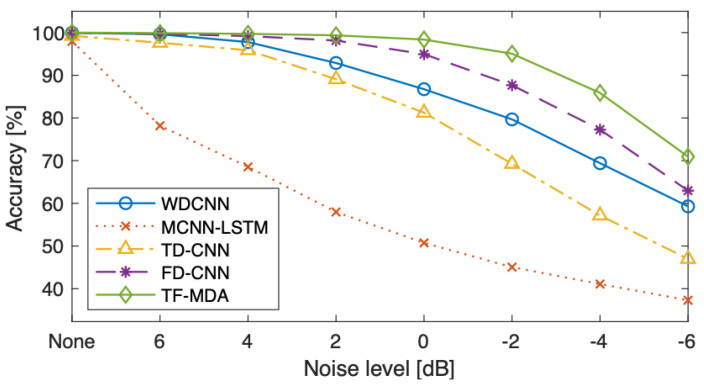
Classification accuracy without and with various noise conditions for different models [%].

The robustness against noisy environment conditions of the proposed model is compared with other models in Table 8. The classification accuracies under conditions with and without noise are exhibited in Figure 7. All models were tested using the datasets with noise of various levels.

The noise robustness can be evaluated as how much the accuracy is maintained when noise is added to signals. Table 8 and Figure 7 confirmed that the proposed model has dropped its performance only by 3% under noise of 0 dB, whereas other compared models dropped the accuracy by at least 14%. Furthermore, the proposed model maintained the highest accuracy with the lowest degradation rate under all SNR conditions, indicating that the proposed model has strong robustness to noisy environment.

##### t–SNE Analysis

**Figure 8:**
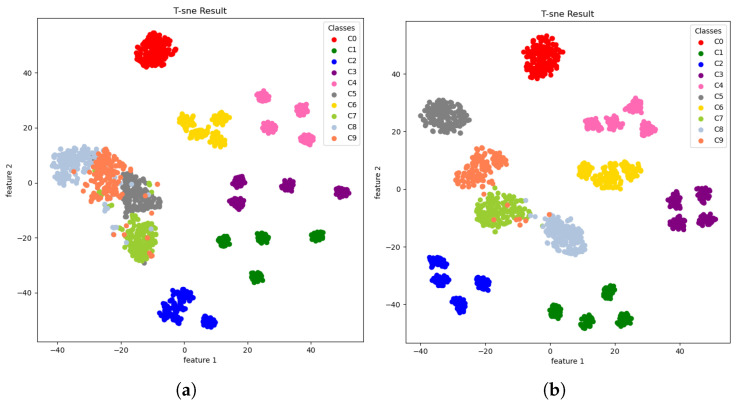
t-SNE: dimension-reduced features extracted from the last convolutional layer of (**a**) TF-MDA and (**b**) TF-MDA (CSA) for test data with SNR 0 dB noise added.

The robustness enhancement of the proposed model is visualized with a t-SNE plot. The feature distributions at the last convolution layer were compared with the plot of the naive model without attention in Figure 8. Both models had satisfactory feature clustering for high separability even with the data corrupted by 0 dB noise. However, TF-MDA+CSA yielded clearer boundaries among different classes, allowing more accurate identification of fault types, as shown in Figure 8b. It can be concluded that adding the attention module to the proposed network improved both the robustness and accuracy in a noisy environment.

### 4.3. Case Study: Paderborn University (PU) Dataset

#### 4.3.1. Dataset Description

The whole experiment was repeated with other open datasets provided by Paderborn University [50]. This is a set of data of normal and faulty bearings captured by an accelerometer with a 64-kHz sampling rate. For each test setting, 20 measurements of 4 s each were recorded. The machinery is a 425 W permanent magnet synchronous motor (PMSM) with the motor specifications shown in Table 9. It was operated under four operating conditions, but only three were selected for this study, summarized in Table 10. The test bearings are 6203 ball-bearing type with their mechanical specifications summarized in Table 11. Using the bearing specifications, the BPFI and BPFO of the bearing can be estimated using Equations (1) and (2), and their values are shown in Table 12. The motor operation speed and fault characteristic frequencies are close to the CWRU dataset used in Case Study 1. However, the sampling frequency was about 5 times different, so the PU dataset was downsampled to 1/5.

The dataset includes both artificial- and real-damaged bearings data, and each is assigned a bearing code. For this study, one normal and eight faulty bearing data under various operating conditions were selected. The fault classes were defined based on bearing fault type, fault level, and fault location, and detailed information is summarized in Table 13. There were a total of 10,260 data samples, and the training, validation, and testing ratios are set at 6:2:2.

#### 4.3.2. Comparison Study

**Figure 9:**
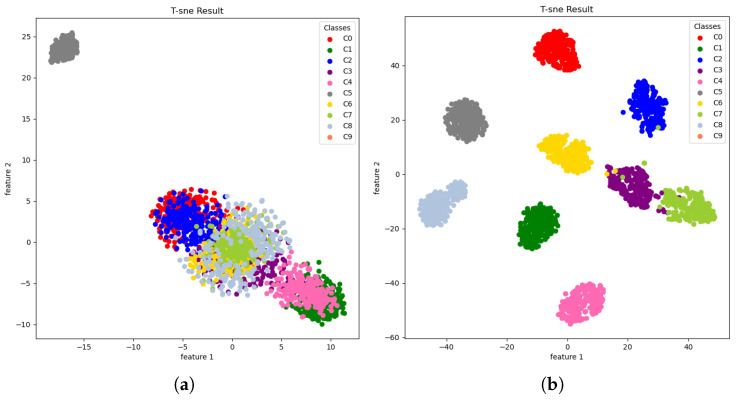
t-SNE: dimension-reduced features extracted from TF-MDA (CSA) for test data of PU dataset with SNR 0 dB noise added: (**a**) first convolutional layer; (**b**) fourth convolutional layer.

Like the CWRU dataset, the proposed model and comparison models are tested under different noise conditions, as shown in Table 14. Under no noise conditions, WDCNN, FD-CNN, and TF-MDA (CSA) showed an accuracy of 100%. As the noise level was increased from 6 dB to −6 dB, the proposed model maintained the highest accuracy with the lowest degradation rate. FD-CNN showed the second-highest performance, even with a noise level of 0 dB. In contrast, other compared models indicated a rapid performance degradation under a noise environment. Figure 9 shows the t-SNE results of the TF-MDA model tested under a noisy environment. The features extracted from the first layer are all mixed up, making them difficult to distinguish except for one class. However, the features extracted from the fourth layer are almost clearly distinguished by class, although there is some confusion due to the noise added to the dataset.

Therefore, it was demonstrated that the proposed model with multi-domain feature fusion and attention modules could extract more useful representations for superior performance in a noisy environment.

## 5. Conclusions

This paper proposed a noise-robust and accurate bearing fault monitoring model based on a time-frequency multi-domain 1D CNN with attention modules. The proposed model uses both the time domain vibration signals and the corresponding frequency spectrum to obtain comprehensive information on fault vibrations for accurate classification. Additionally, to enhance the classification robustness in high-noise environments, a series of channel and spatial attention modules were added to the frequency feature extraction. Experimental results confirmed that applying the attention modules improved the noise robustness by approximately 8% compared with the case without it. The proposed model achieved 100% accuracy when tested in a noise-free environment, and showed a high accuracy of 84.75% at a noise condition with SNR −6 dB. The effectiveness of attention modules was also visualized with t-SNE plots. Furthermore, the accuracy of the proposed model was compared with similar bearing fault models using the CWRU dataset and Paderborn dataset with Gaussian noise added. Results indicated that the proposed model exhibited the highest accuracy for all the noise levels in the range of −6 to 6 dB. However, the noise added to the dataset for this study is artificial Gaussian white noise. Real industrial conditions have various types of noise such as resonance, rattle noise of gear, and noise from other machinery. In addition, in real industrial sites, multiple types of bearing faults may occur simultaneously, but the dataset used in this study contains only single-fault bearing data. In future work, the proposed model should be tested and improved using the dataset obtained from various industry sites.

## Figures and Tables

**Table 1:** Accuracy in cases of placing an attention layer behind each convolution layer of FD-CNN.

Conv. Layer	SNR [dB]							
	None	6	4	2	0	−2	−4	−6
1st layer	**100.00**	**99.83**	**99.42**	**98.37**	**95.99**	89.71	**79.36**	**64.77**
2nd layer	**100.00**	99.65	99.13	97.79	95.06	89.13	76.80	61.16
3rd layer	**100.00**	99.53	99.07	97.91	95.35	**90.87**	78.84	62.85
4th layer	**100.00**	99.77	99.36	98.26	95.93	90.06	77.73	60.17
5th layer	**100.00**	99.77	99.36	98.31	95.41	90.23	79.24	62.56

**Table 2:** Architecture of TF-MDA.

TD-CNN					FD-CNN				
Layer	Kernel Size /Stride	No. of Kernels	Output Shape	Padding	Layer	Kernel Size /Stride	No. of Kernels	Output Shape	Padding
Conv1	256/5	4	154 × 4	-	Conv1	256/5	4	58 × 4	2
Conv2	15/3	8	47 × 8	-	CBAM	64/1	4	58 × 4	-
Conv3	7/2	16	21 × 16	-	Conv2	5/2	8	29 × 8	2
Conv4	7/2	32	8 × 32	-	Conv3	5/2	16	15 × 16	2
Conv5	3/1	64	6 × 64	-	Conv4	5/2	32	8 × 32	2
GAP	-	-	64 × 1	-	Conv5	5/2	64	4 × 64	2
				-	GAP	-	-	64 × 1	-
DFMLP									
	Layer		Input shape			Output shape			
	FC1: Domain fusion		128 × 1			64 × 1			
	FC2		64 × 1			32 × 1			
	FC3		32 × 1			10 × 1			

**Table 3:** Defect frequencies at different fault locations of CWRU bearing. $f_{r}$ is the shaft rotating frequency.

	Inner Race	Outer Race	Ball
Defect freq. [Hz]	$5.42\times f_{r}$	$3.58\times f_{r}$	$4.71\times f_{r}$

**Table 4:** Classes of the CWRU dataset based on fault types.

Class No.	Fault Location	Fault Size [mils]	Working Loads [HP]	Train Samples		Valid Samples		Test Samples	
C0	Normal	-	0	129	516	43	172	43	172
			1	129		43		43	
			2	129		43		43	
			3	129		43		43	
C1	Inner race fault	7	0	129	516	43	172	43	172
			1	129		43		43	
			2	129		43		43	
			3	129		43		43	
C2	Inner race fault	14	0	129	516	43	172	43	172
			1	129		43		43	
			2	129		43		43	
			3	129		43		43	
C3	Inner race fault	21	0	129	516	43	172	43	172
			1	129		43		43	
			2	129		43		43	
			3	129		43		43	
C4	Outer race fault	7	0	129	516	43	172	43	172
			1	129		43		43	
			2	129		43		43	
			3	129		43		43	
C5	Outer race fault	14	0	129	516	43	172	43	172
			1	129		43		43	
			2	129		43		43	
			3	129		43		43	
C6	Outer race fault	21	0	129	516	43	172	43	172
			1	129		43		43	
			2	129		43		43	
			3	129		43		43	
C7	Ball fault	7	0	129	516	43	172	43	172
			1	129		43		43	
			2	129		43		43	
			3	129		43		43	
C8	Ball fault	14	0	129	516	43	172	43	172
			1	129		43		43	
			2	129		43		43	
			3	129		43		43	
C9	Ball fault	21	0	129	516	43	172	43	172
			1	129		43		43	
			2	129		43		43	
			3	129		43		43	

**Table 5:** Accuracy under conditions without noise and the number of parameters of attention module inserted into each model.

Model	Attention Module	No. of Params	Accuracy [%]
TF-MD+NA	None	38,162	99.94
TF-MDA (SA)	Spatial attention only	38,293	**100**
TF-MDA (CA)	Channel attention only	38,188	99.94
TF-MDA (CSA)	Spatial–Channel Attention	38,319	**100**

**Table 6:** Classification accuracies of different models under condition without noise.

Model	No. of Params	Accuracy [%]
WDCNN [49]	38,162	99.95
MCNN–LSTM [19]	73,480	97.97
TD–CNN	14,910	99.24
FD–CNN	17,406	99.94
TF–MDA (CSA)	38,319	**100**

**Table 7:** Classification accuracy under various noise conditions for different attention modules [%].

Model Name	SNR [dB]						
	6	4	2	0	−2	−4	−6
TF–MD+NA	**99.88**	99.65	98.78	96.74	90.81	81.86	66.40
TF–MDA (SA)	99.83	99.59	98.84	96.05	90.93	77.44	64.13
TF–MDA (CA)	**99.88**	**99.77**	99.13	96.86	90.99	80.76	66.63
TF–MDA (CSA)	**99.88**	99.71	**99.36**	**98.37**	**95.06**	**85.87**	**70.93**

**Table 8:** Classification accuracies under various noise conditions of different models [%].

Model	SNR [dB]							No. of Params
	6	4	2	0	−2	−4	−6	
WDCNN [49]	99.62	97.77	92.88	86.74	79.67	69.40	59.29	54,510
MCNN–LSTM [19]	78.18	68.54	57.97	50.73	45.05	41.09	37.29	73,480
TD–CNN	97.62	95.87	89.07	81.22	69.24	57.15	46.98	14,910
FD–CNN	99.71	99.19	98.14	94.94	87.67	77.27	62.97	17,406
TF–MDA (CSA)	**99.88**	**99.71**	**99.36**	**98.37**	**95.06**	**85.87**	**70.93**	38,319

**Table 9:** Motor specification of PU dataset.

Description	Nominal Torque	Nominal Speed	Nominal Current	Pole Pair
Value	1.35 [Nm]	3000 [rpm]	2.3 [A]	4

**Table 10:** Operating conditions of PU dataset.

No.	Rotational Speed [rpm]	Load Torque [Nm]	Radial Force [N]
OC 1	1500	0.7	1000
OC 2	1500	0.1	1000
OC 3	1500	0.7	400

**Table 11:** Specification of bearing of PU dataset.

Notation	Meaning	Value
*D*	Pitch circle diameter	28.55 [mm]
*d*	Rolling element diameter	6.75 [mm]
*Z*	No. of rolling elements	8
α	Contact angle	0°

**Table 12:** Fault characteristic frequency of PU dataset.

	Inner Race	Outer Race
Defect freq. [Hz]	$4.95\times f_{r}$	$3.05\times f_{r}$

**Table 13:** Bearing fault classes of PU dataset based on fault type.

Class No.	Bearing Code	Fault Generation	Damage Method /Fault Type	Fault Location	Fault Level	Operating Condition
C0	K001	Normal		-	-	OC 1, 2, 3
C1	KI01	Artificial	EDM	Inner race fault	1	
C2	KI07	Artificial	electric engraver	Inner race fault	2	
C3	KI17	Real	fatigue	Inner race fault	1	
C4	KI18	Real	fatigue	Inner race fault	2	
C5	KA01	Artificial	EDM	outer race fault	1	
C6	KA03	Artificial	electric engraver	outer race fault	2	
C7	KA15	Real	Plastic deform	outer race fault	1	
C8	KA16	Real	fatigue	outer race fault	2	

**Table 14:** Classification accuracies under various noise conditions of different models trained with PU dataset [%].

Model	SNR [dB]							
	None	6	4	2	0	−2	−4	−6
WDCNN [49]	**100.00**	99.37	96.15	87.37	79.56	73.04	65.70	58.07
MCNN-LSTM [19]	97.36	70.53	60.58	51.72	43.89	37.57	31.85	28.25
TD-CNN	98.93	94.30	87.72	77.78	65.79	57.46	50.97	47.17
FD-CNN	**100.00**	**99.95**	**99.90**	99.32	97.90	94.20	85.28	69.83
TF-MDA (CSA)	**100.00**	**99.95**	**99.90**	**99.61**	**98.59**	**96.49**	**91.72**	**84.75**

## Data Availability

The data presented in this study are available upon request from the corresponding author.
